# Supplementary material for: Destruction of Lysozyme Amyloid Fibrils Induced by Magnetoferritin and Reconstructed Ferritin
Source: Int J Mol Sci. 2022 Nov 11;23(22):13926. doi: 10.3390/ijms232213926 (PMC9696235; doi:10.3390/ijms232213926)
Supplement: Supplementary file 1 [file ijms-23-13926-s001.zip › figures_supplementary.pdf]

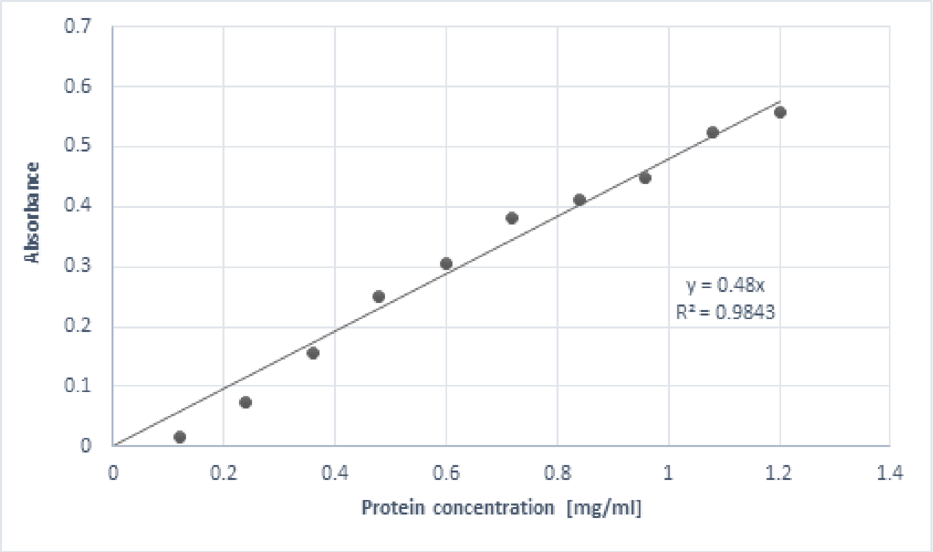

Figure S1a. Calibration curve for determination of protein concentration using Bradford assay.

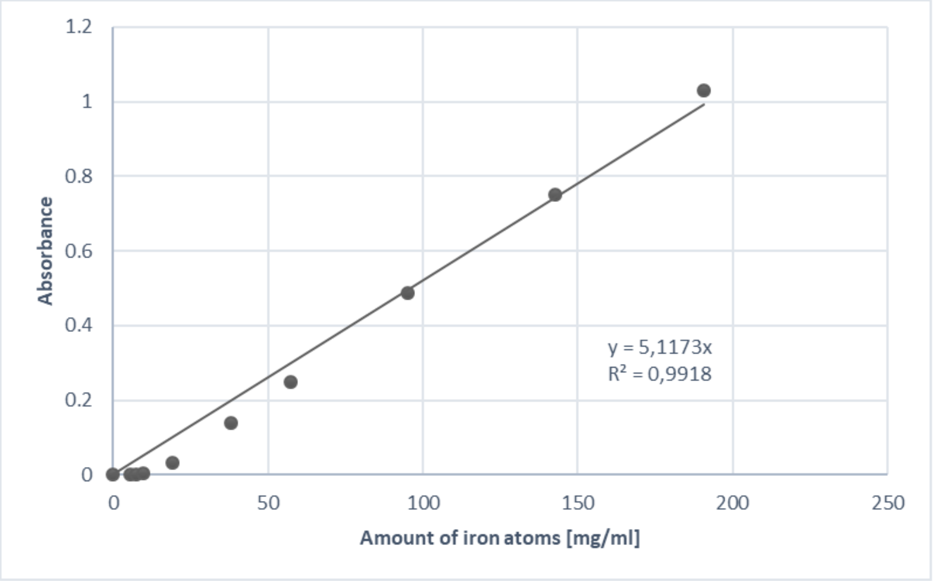

Figure S1b. Calibration curve for determination of the number of iron atoms.

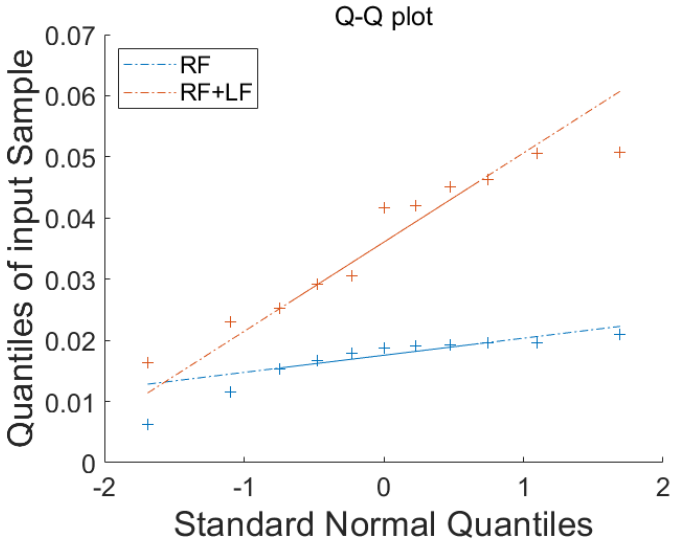

Figure S2a. Q-Q plot of iron release from reconstructed ferritin after interaction with lysozyme amyloid fibrils.

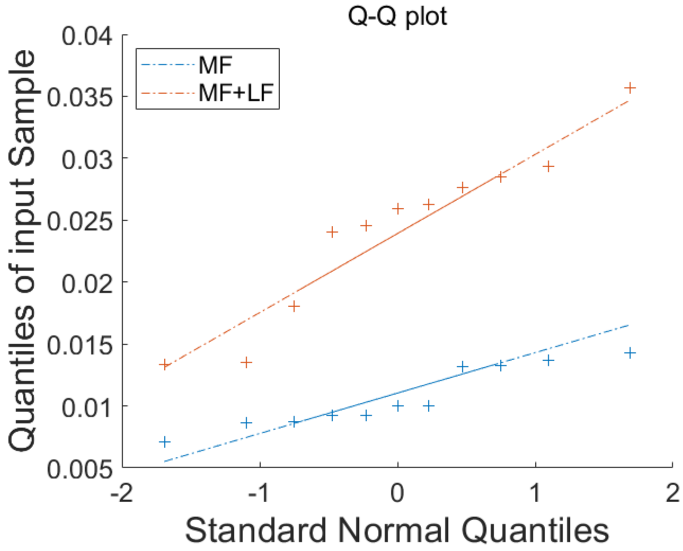

Figure S2b. Q-Q plot of iron release from magnetoferritin after interaction with lysozyme amyloid fibrils.

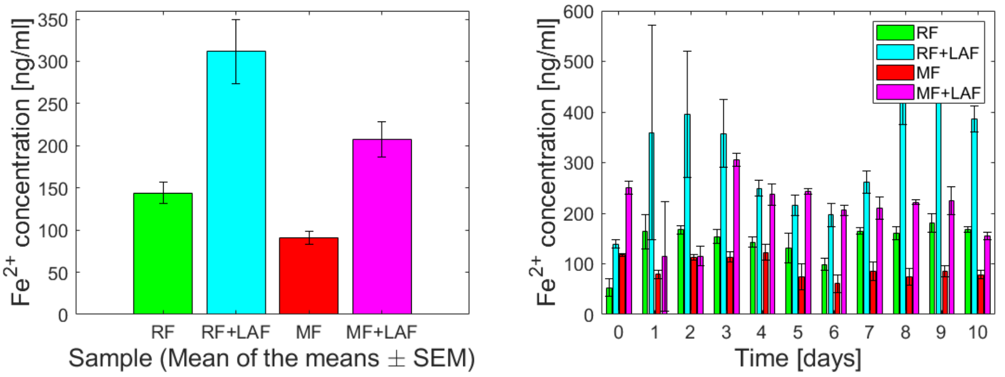

Figure S3. (LEFT) Daily release of ferrous ions (mean ± SD) from ferritin's derivatives. (RIGHT) Average (mean of the means ± SEM) of overall released ferrous ions from ferritin's derivatives per day.

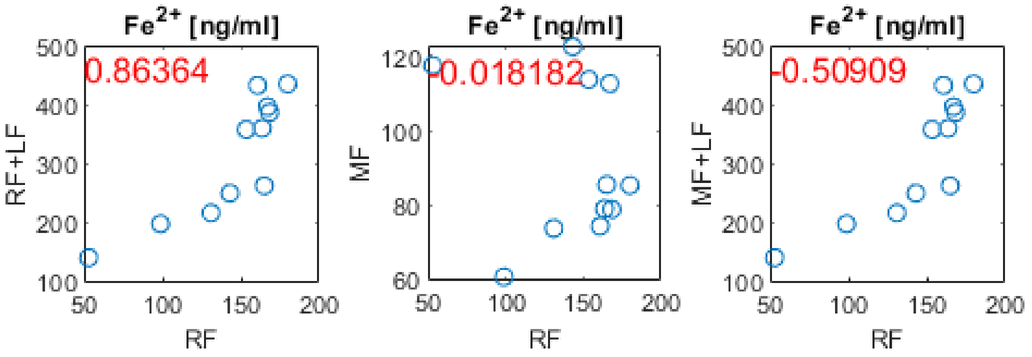

Figure S4a. Spearman correlation of iron release from ferritin derivatives and after their interaction with lysozyme amyloid fibrils.

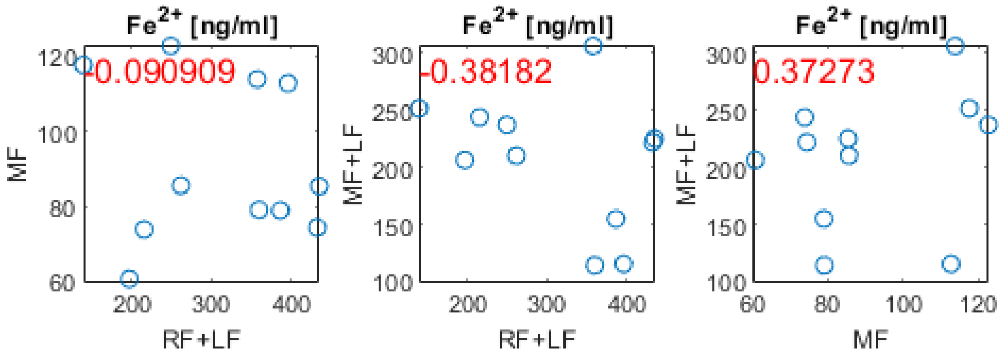

Figure S4b. Spearman correlation of iron release from ferritin derivatives and after their interaction with lysozyme amyloid fibrils.

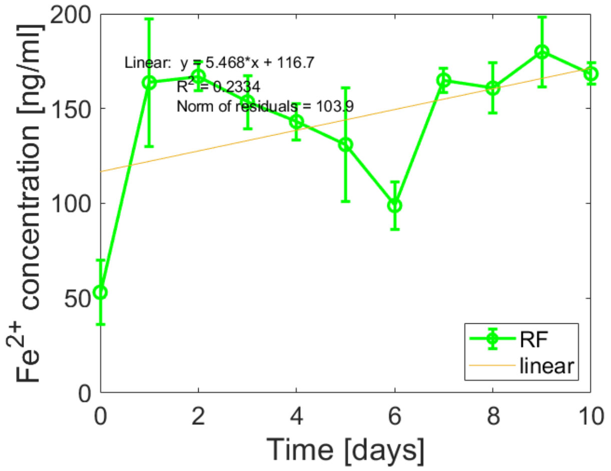

Figure S5a. Regression analysis of iron release from reconstructed ferritin.

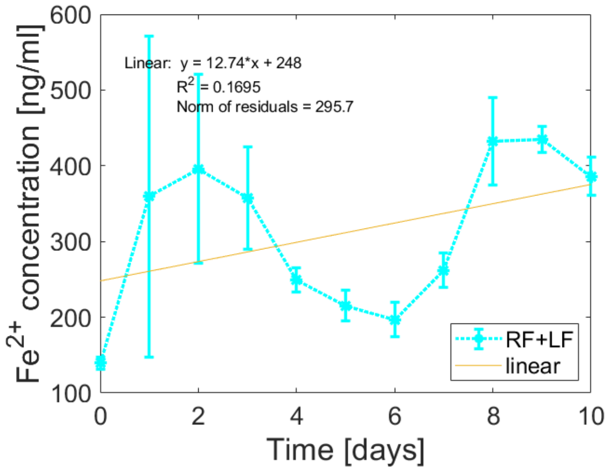

Figure S5b. Regression analysis of iron release from reconstructed ferritin after interaction with lysozyme amyloid fibrils.

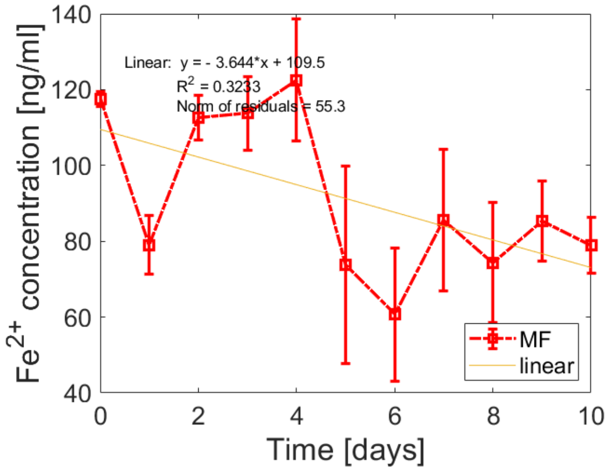

Figure S5c. Regression analysis of iron release from magnetoferritin.

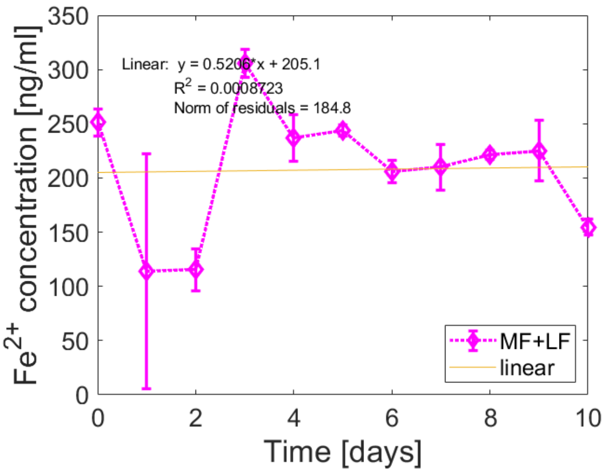

Figure S5d. Regression analysis of iron release from magnetoferritin after interaction with lysozyme amyloid fibrils.
